# Defect Detection in Steel Using a Hybrid Attention Network

**DOI:** 10.3390/s23156982

**Published:** 2023-08-06

**Authors:** Mudan Zhou, Wentao Lu, Jingbo Xia, Yuhao Wang

**Affiliations:** 1 School of Information Science & Technology, Xiamen University Tan Kah Kee College, Zhangzhou 363105, China; 2 School of Electronic Science and Engineering, Xiamen University, Xiamen 361005, China

**Keywords:** defect detection, deep learning, steel surfaces, ASFF, CIOU, mAP, SSD, EfficientNet, YOLOV3, YOLOV5

## Abstract

Defect detection in steel surface focuses on accurately identifying and precisely locating defects on the surface of steel materials. Methods of defect detection with deep learning have gained significant attention in research. Existing algorithms can achieve satisfactory results, but the accuracy of defect detection still needs to be improved. Aiming at this issue, a hybrid attention network is proposed in this paper. Firstly, a CBAM attention module is used to enhance the model’s ability to learn effective features. Secondly, an adaptively spatial feature fusion (ASFF) module is used to improve the accuracy by extracting multi-scale information of defects. Finally, the CIOU algorithm is introduced to optimize the training loss of the baseline model. The experimental results show that the performance of our method in this work is superior on the NEU-DET dataset, with an 8.34% improvement in mAP. Compared with major algorithms of object detection such as SSD, EfficientNet, YOLOV3, and YOLOV5, the mAP was improved by 16.36%, 41.68%, 20.79%, and 13.96%, respectively. This demonstrates that the mAP of our proposed method is higher than other major algorithms.

## 1. Introduction

Steel is a crucial raw material for various industries including automobile manufacturing, aerospace equipment manufacturing, and infrastructure projects like bridges. It has significantly contributed to the economic development and technological progress of modern society. However, some issues such as equipment failure, operational errors, and transportation damage can generate multiple surface imperfections and defects during the production and transportation of these raw materials. For example, the steel surface can produce cracks, scratches, patches, punches, indentations, and other defects, which can significantly impact the safety and reliability of industrial manufacturing. Therefore, research on surface defect detection is particularly important.

With the continuous development of machine vision and computer technology, the methods for detecting the surface defects of metal have evolved from manual visual inspection to automated machine inspection. Liu et al. [1] utilized a multivariate discriminant function to compute the parameters of a statistical histogram for extracting features in cold-rolled steel strips. Similarly, Luo et al. [2] employed the GCLBP method, which utilizes pattern and frequency information of LBP, to detect defects on metal surfaces. Ai et al. [3] employed statistical features derived from the amplitude spectrum obtained through the Fourier transform to detect cracks on steel plate surfaces. In a different approach, Medina et al. [4] detected defects in steel coils using Gabor filters in both spatial and frequency domains. There was a poor anti-interference ability of these manual methods of feature extraction with complex parameter calculations. In summary, achieving high performance in defect detection using conventional methods is challenging.

In recent years, there has been significant progress in the research of deep learning methods in computer vision. Specifically, detection methods using deep learning can be categorized into two main types based on the network structure: one-stage detection networks and two-stage detection networks. In a two-stage detection network, a region generation network is employed to analyze the approximate locations of feature points and generate multiple regions of interest. Subsequently, effective features are extracted from these regions of interest to achieve precise object localization. Typical two-stage networks of object detection include RCNN [5], Fast-RCNN [6], Faster-RCNN [7], and Cascade-RCNN [8], all of which have demonstrated good performance. On the other hand, in one-stage networks, the results of object detection are directly output using an end-to-end format. The one-stage network achieves faster detection, but it sacrifices some accuracy compared to the two-stage network. Some popular one-stage networks for object detection are YOLO [9,10,11,12,13], SSD [14], FCOS [15], and ATSS [16]. In the context of defect detection on metal surfaces, Yu [17] proposed SD-Net, a method that utilized Yolov3 as the baseline, which combined a Res-Net with an improved spatial pyramid module. Ma et al. [18] developed a two-channel attention algorithm using Yolov4 for the surface inspection of aluminum strips. In a similar vein, Guo et al. [19] introduced a transformer module based on Yolov5, capable of extracting global features and achieving high accuracy of object detection.

The current focus of research in object detection is to improve accuracy by increasing the depth and width of the networks [20,21]. These methods have been able to achieve better detection results for object detection, but it is difficult to maintain a high level of accuracy for small-scale defective samples, especially for the defects in steel structures. A new one-stage network that aims to improve the accuracy of defect detection is proposed in this paper. Using YOLOX-S as the baseline algorithm, it is a single-stage network of defect detection based on an approach of hybrid domain attention and adaptive spatial weight in feature fusion. For the feature extraction network, CBAM, an attention module is proposed to enhance the ability to learn effective features. For the feature fusion network, ASFF, the adaptively spatial feature fusion module is used to improve its accuracy, which extracts multi-scale information of defects. At the moment of training the last model, the CIOU algorithm is introduced to optimize the training loss of the baseline model.

In summary, the main innovations and contributions in this work can be categorized into the following three areas.

- Introduce the CBAM to Backbone to enhance the learning capability of the whole network.
- Take advantage of ASFF in the Neck section to enhance the extraction of multi-scale semantic information of defects.
- The loss function of CIOU is incorporated to address the issue of poor generalization and enhance the model training process.

## 2. Related Work

### 2.1. Defect Detection Based on Traditional Machine Vision

Traditional machine vision technology has been previously developed and it has been applied in the field of digital image processing for quite a long time. There are a large number of published related papers. The traditional methods are listed below:

- **Background Phase Subtraction:** Subtracts the background image without defects estimated or calculated in advance from the background image, leaving a residual image containing defects and random noise.
- **Traditional methods of image processing using filtering techniques.** Guan et al. used a non-static algorithm for batch-by-batch detection [22]. With the help of the significance of textile defects, this algorithm was used to detect defects in textiles located in the color space of HSV. It estimated the defects of different textiles better and also had good generalization and generality. To study randomly textured color images, Shafarenko et al. proposed a measurement of color similarity based on the watershed algorithm [23], which achieved automatic detection of defects on surfaces in randomly textured color images based on color, texture, edges, and other features of different images. Hoang et al. investigated how to detect defects on leather surfaces [24]. Firstly, the corrosion operation was used, then automatic image segmentation was performed using the method of OTSu. Finally, a clustering algorithm was used to classify various defects, which was based on the Euclidean distance, and the experimental results showed the effectiveness of this method.
- **Feature detection based on manual features.** Furthermore, these features were utilized to train the classifier of machine learning and achieved the final defect detection.

In fact, there is a great deal of intersection between these methods, and several traditional methods may be used simultaneously. In general, traditional methods can only detect defects under certain conditions, such as having a certain size or a distinct defect profile with strong contrast and low noise under the conditions of certain lighting. Machine learning has a certain robustness, but the disadvantages of artificial features are weak characterization and poor adaptability. Thus, algorithms of machine learning do not learn the features that need to be detected well.

### 2.2. Defect Detection Based on Deep Learning

Compared with the technology of traditional machine vision, deep learning has only been widely used in recent years but has achieved outstanding results. The development of big data and the continuous improvement in computer performance provide a broader development platform for the application of deep learning in the technology of nondestructive detection. CNNs rely on large-scale training datasets and reasonable supervised learning algorithms. Compared with manually designed features, they can obtain more essential features, which can greatly improve the effect of detection of the algorithm.

Chen et al. investigated the automated defect detection of fasteners on cable support units [25]. In this work, the cascade of the whole system consisted of two detectors, which were based on YOLO and SSD. They were used to locate the cantilever joints and their fasteners sequentially, and the structure also contained a classifier to determine which class of defects the fasteners belonged to. When using SSD for localizing cantilever joints, Conv3×3, a larger convolution layer, was added to obtain more shallow information about images since the input images contained many small-scale defects. This improvement resulted in a definite increase in accuracy compared to the original model.

Tao et al. discussed the automatic defect detection of metals using a two-stage network [26], which achieved precise localization and classification of defects appearing in the input images captured from industrial environments. In this work, CASAE, the novel architecture of a cascaded auto-encoder, was designed to segment and localize defects. It is well known that an auto-encoder consists of encoder and decoder networks. The input image is transformed into a feature map, which was more favorable for feature extraction and feature matching by the encoder. The pixel-level labels are adjusted by merging semantic and contextual information from a series of feature maps by the decoder. Based on semantic segmentation, the input image is transformed into a mask by the cascaded auto-encoder, while the defective regions after segmentation are classified into the specific classes to which they belonged through a convolution neural network. The experiments showed that the AE (auto-encoder) model outperformed the full convolution network, and CASAE could also improve the value of IOU. Due to considering different sizes of receptive fields, the four convolution layers in the encoder of the AE network had to be replaced by dilated convolution. This demonstrated that dilated convolution was important for learning the features of defects at different scales.

Lan et al. used an improved algorithm of Faster R-CNN to detect defects in the images from UAVs [27]. This improvement was divided into two parts: Firstly, the operations of cropping and resizing were used to replace the pooling layer in the region of interest. Secondly, to better recognize small-scale objects, all small-region candidate modules in the improved network were retained and the regions with scales larger than 16 pixels in the original network were removed. The following conclusions could be deduced from this experiment: due to the increase in the size of the input image, the model achieves a better performance. With the dataset in this study, the mAP using VGG16 was 81.11%, and the mAP for the improved method was only 67.92%. This was due to overfitting of the model caused by unbalanced samples.

Yang proposed a CNN-based steel surface defect detection algorithm for problems such as the low recognition rate of steel surface defects [28]. Zhang combined a residual network and Faster R-CNN to detect steel surface defects [29]. This improved the detection accuracy by a small margin compared to the traditional methods, but its computational and parametric quantities were very large and the algorithm was poor in real time, which was not favorable for industrial use.

Cheng et al. proposed an improved defect detection method based on YOLOv3 [30], which was used to solve the problem of the small size and unclear features of defects on metal surfaces, with good detection results. Shun et al. used the YOLOv5 network for the detection of steel surface defects and improved the detection accuracy by 11.9% compared to YOLOv4 [31].

## 3. Baseline

**Figure 1:**
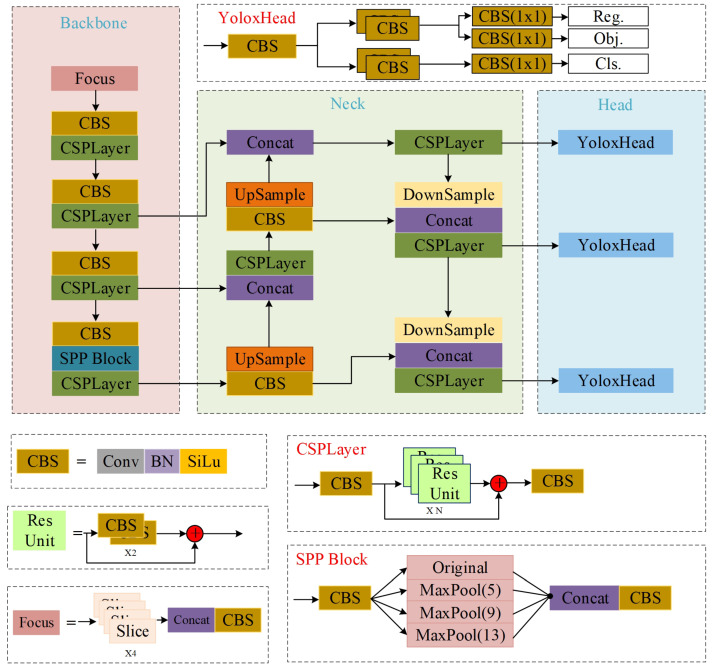
The construction of YOLOX-S.

YOLOX [13] is a prominent network in object detection within the series of YOLO, known for its optimal combination of accuracy and speed. Comprising three key components—Backbone, Neck, and Yolo-Head—the structural framework of YOLOX is illustrated in Figure 1.

### 3.1. Feature Extraction Network

As shown in Figure 1, CSP-Dark-Net is used as the backbone in the network of feature extraction. In this study, a Focus structure was employed to downsample the images, thereby reducing the complexity of the network and the number of computational parameters. By increasing the number of channels of the feature map and reducing the image scale in Focus, the model was able to effectively preserve semantic information. Additionally, CSP-Layer, a cross-stage local network, was introduced into the whole stage, splitting the gradient streams to propagate through different paths. This approach combined various types of gradients in the feature extraction network, resulting in a reduction in the number of network parameters. Finally, SPP, a Spatial Pyramid Pooling module, was used to enhance the perceptual field of the feature layer in the network.

### 3.2. Feature Fusion Network

In YOLOX-S, the Path Aggregation Feature Pyramid Network (PAFPN) was utilized to merge the features generated by the backbone network to extract deep information more comprehensively. Initially, the features of various dimensions and scales were up-sampled to allow the semantic information to flow from deep features to shallow ones. Subsequently, the features were down-sampled to make the characterization information flow from shallow features to deep ones. At this stage, the feature map was sampled twice, resulting in relatively complete semantic and representational features. The process of the PAFPN enhanced the defect features by providing them with meaningful surface characteristics.

### 3.3. YOLOX-Head

In object detection, the module of YOLOX-Head is responsible for localizing and classifying valid targets in the feature map. This is accomplished by localizing and regressing the feature maps using effective features generated by the feature fusion network. As depicted in Figure 1, the Baseline introduces the decoupling head approach, where the feature maps of different channels are unified through a 3×3 convolution layer. The decoupling head is divided into two parallel branches, both utilizing the 3×3 convolution layer for target localization and classification calculations. Finally, the prediction results are obtained through the operation of a 1×1 convolution layer.

## 4. Our Methods

**Figure 2:**
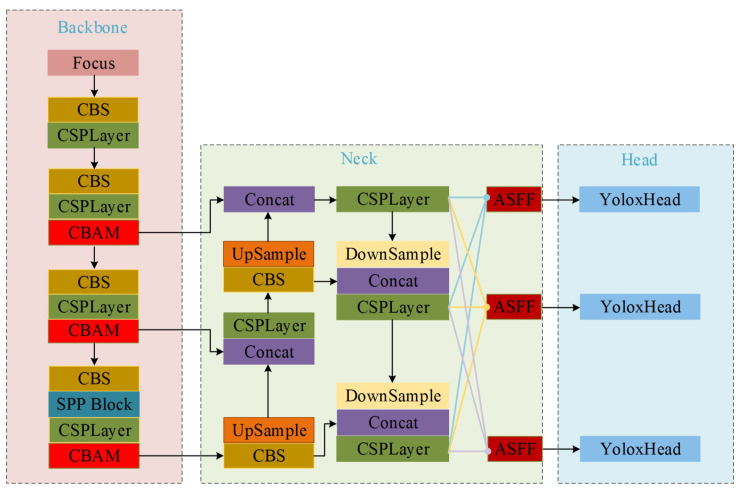
The structure of the improved YOLOX-S.

In this paper, YOLOX-S is used as the baseline for training on a dataset of the structure of steel. Upon analysis, it was discovered that the model a exhibits low detection accuracy and struggles with identifying small-scale defects in the NEU-DET dataset. To address these problems in the defect detection of steel surfaces, an improved algorithm was proposed in this paper. It illustrates three primary improvements based on the baseline in Figure 2.

- CBAM is introduced into the Backbone to enhance the learning capability of the whole network. CBAM is a hybrid attention mechanism that combines the advantages of the attention of the bath channel and the spatial domain for feature detection. It improves the ability of the model to extract deep features.
- ASFF is taken advantage of in the Neck section to enhance the extraction of multi-scale semantic information of defects. ASFF is implemented in the feature fusion network, which allows the model to learn the semantic information from different feature layers and assign appropriate weights to each feature map layer. It can improve the accuracy of the network detection.
- The loss function of CIOU is incorporated to address the issue of poor generalization and enhance the model training process.

### 4.1. Convolution Block Attention Module

**Figure 3:**
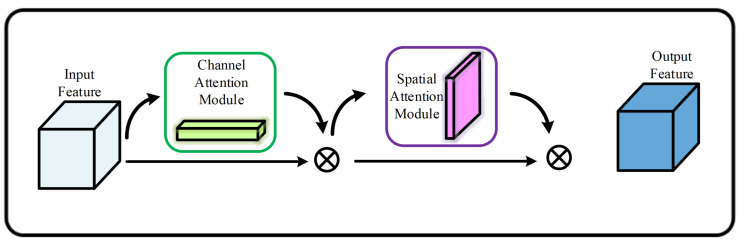
CBAM Module.

The attentional mechanism is a method of adapting the parameters of a network that mimics human visual perception by learning and focusing on specific parts. CBAM [32] is a simple and effective attention module for feedforward convolutional neural networks, as shown in Figure 3. CBAM contains two modules, namely a Channel Attention Module (CAM) and a Spatial Attention Module (SAM). For an input feature map, CBAM will infer the attention map along two independent dimensions in turn and then multiply the attention map by the input feature map for adaptive feature optimization. CBAM has a low number of parameters, so it can be added to all stages of convolutional neural networks for end-to-end training.

**Figure 4:**
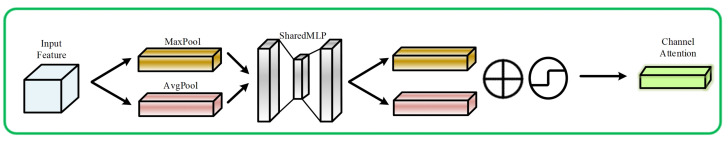
The structure of CAM.

The structure of CAM is depicted in Figure 4. It compresses the feature map in the spatial dimension to obtain a thought vector and then correlates it. When compressing in the spatial dimension, not only Average Pooling but also Max Pooling are considered. Average Pooling and Max Pooling can be used to aggregate the spatial information of feature maps sent to a shared network, compress the spatial dimensions of the input feature maps, and merge them element-by-element to produce channel attention maps. In the case of a map alone, attention is channelled; the focus is on what on that map is of importance. Average Pooling has feedback for every pixel point in the feature maps, whereas Max Pooling has feedback for the gradient only when the response is largest in the feature maps when computing the gradient of backward propagation. The specific implementation of CAM can be expressed as:

(1) $$M_{c}=\sigma \left(MLP\left(AvgPool\left(F\right)\right)\right)+MLP\left(MaxPool\left(F\right)\right)$$

**Figure 5:**
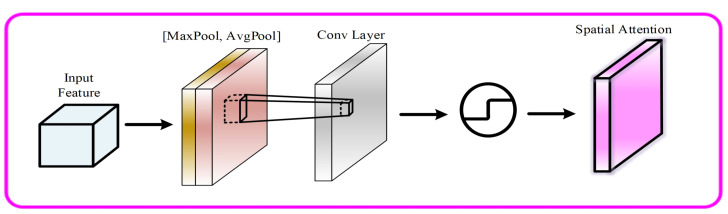
The structure of SAM.

As depicted in Figure 5, SAM is a module of channel compression that utilizes Average Pooling and Max Pooling in the channel dimension. It takes the feature map output from channel attention as the input feature map for this module. Firstly, global Max Pooling and global Average Pooling are performed for the channel’s features, and then the results are concatenated based on the channel. Secondly, after a convolution operation, the dimensions of the feature are reduced to one channel, and then the feature in spatial attention is generated by the sigmoid function. At last, the feature is multiplied with the input feature of this module to obtain the final generated feature.

Similarly, the spatial attention module (SAM) is a compression of the channels with Average Pooling and Max Pooling in the channel dimension, respectively. The operation of Max Pooling extracts the maximum value of the channel, and the number of extractions is the product of the height and width of the feature. The operation of Average Pooling extracts the average value of the channel, and the number of times it is extracted is also the height multiplied by the width. Then, previously extracted feature maps are merged to obtain a two-channel feature map. It can be expressed as:

(2) $$M_{s}\left(F\right)=\sigma \left(f^{7\times 7}\left(\left[AvgPool(F),MaxPool(F)\right]\right)\right)$$

### 4.2. Adaptively Spatial Feature Fusion

**Figure 6:**
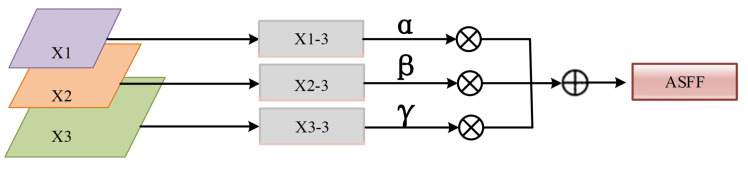
The process of ASFF.

In the stage of feature fusion, achieving effective fusion of smaller-scale features in the dataset is challenging for the PAFPN. This can lead to a low accuracy of detection and a high miss rate of defect features. To address these problems, ASFF, adaptively spatial feature fusion [33], is proposed to optimize the Neck structure in this paper. It makes the network adaptively learn the spatial weights of features in each feature map. The structure of ASFF is illustrated in Figure 6.

It takes three different scales of effective features $X^{1}$, $X^{2}$, and $X^{3}$ from the PAFPN as input in ASFF. By applying constant scaling, three features with equal dimensions $X^{1\to 3}$, $X^{2\to 3}$, and $X^{3\to 3}$ are obtained, generating semantic information at different scales. These features are then combined with the corresponding adaptive weights to achieve adaptive fusion. The method for adaptive feature fusion of feature maps is shown as:

(3) $$Y_{i,j}^{3}=\alpha_{i,j}^{3}X^{1\to 3}+\beta_{i,j}^{3}X^{2\to 3}+\gamma_{i,j}^{3}X^{3\to 3}$$

### 4.3. Loss Function of CIOU

The loss function of the baseline comprises three components: $Loss_{cls}$, $Loss_{box}$, and $Loss_{GIOU}$. The approach of GIOU is employed for the loss of boundary regression, which is calculated as shown in Equation (4). In this equation, *C* represents the minimum rectangular area formed by the real frame and the predicted frame. *A* and *B* denote the area of the real frame and the predicted frame, respectively, and *D* represents the intersection ratio of the real frame and the predicted frame.

(4) $$Loss_{GIOU}=1-IOU\left(A,B\right)+\frac{\left|C|-|A\cup B\right|}{\left|C\right|}$$

**Figure 7:**
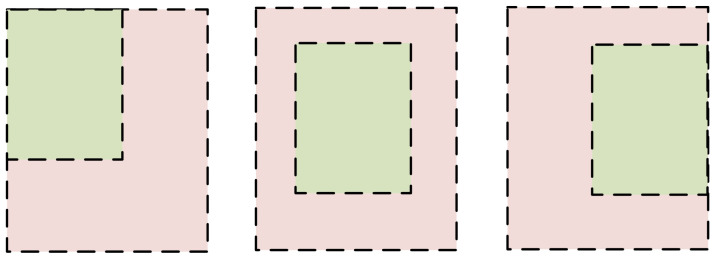
The disadvantages of GIOU. We can notice that the values of the IOU for the three different scenarios in this figure are the same, but this is something we don’t want to see. This is the disadvantage of the GIOU.

According to Equation (4), the calculation of GIOU also has certain limitations. In cases where the real frame and the predicted frame have an inclusion relationship but different positions, the values of GIOU remain the same, as illustrated in Figure 7.

For this problem, DIOU [34], a new loss function of the bounding box, was proposed, which is calculated as shown in Equation (5).

(5) $$Loss_{DIOU}=1-IOU\left(A,B\right)+\frac{\rho^{2}\left(A,B\right)}{c^{2}}$$

**Figure 8:**
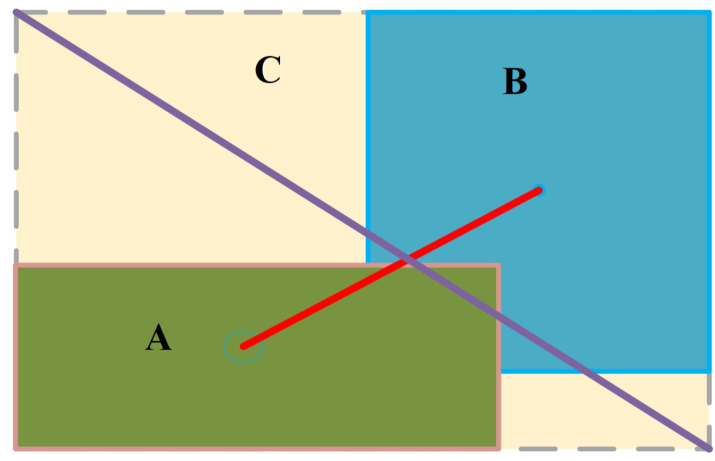
Diagram of DIOU.

As shown in Figure 8, $\rho^{2}\left(A,B\right)$ denotes the Euclidean distance from the center of the bounding box and *c* denotes the diagonal of rectangle *C*.

It considers the distance between the prediction frame and the real frame, the overlap rate, and the scale problem for the metric of DIOU. This leads to more stable boundary regression and optimizes model training.

According to Equation (5), it is determined that $Loss_{DIOU}$ is inversely proportional to the diagonal of rectangle *C*. When the bounding box has a fixed Euclidean distance, a larger diagonal will result in a smaller value, which can affect the convergence of the loss of boundary regression. In this paper, we proposed the loss of CIOU, which introduces an impact factor to the penalty term of DIOU. The procedure of CIOU is expressed as follows:

(6) $$Loss_{CIOU}=1-IOU\left(A,B\right)+\frac{\rho^{2}\left(A,B\right)}{c^{2}}+\alpha \upsilon$$

(7) $$\upsilon =\frac{4}{\pi^{2}}{\left(arctan\frac{w}{h}-arctan\frac{w^{gt}}{h^{gt}}\right)}^{2}$$

(8) $$\alpha =\frac{\upsilon }{1-IOU(A,B)+\upsilon }$$

In the given equations, $\frac{w}{h}$ represents the aspect ratio of the predicted box, $\frac{w^{gt}}{h^{gt}}$ represents the aspect ratio of the real box, α is the weighting factor, and υ is used to measure the consistency of the relative proportions of the two rectangular boxes. υ takes into account the relative scales of the two rectangular boxes based on DIOU, thereby enhancing the accuracy of regression.

## 5. Experimental Results and Analysis

### 5.1. Dataset

**Figure 9:**
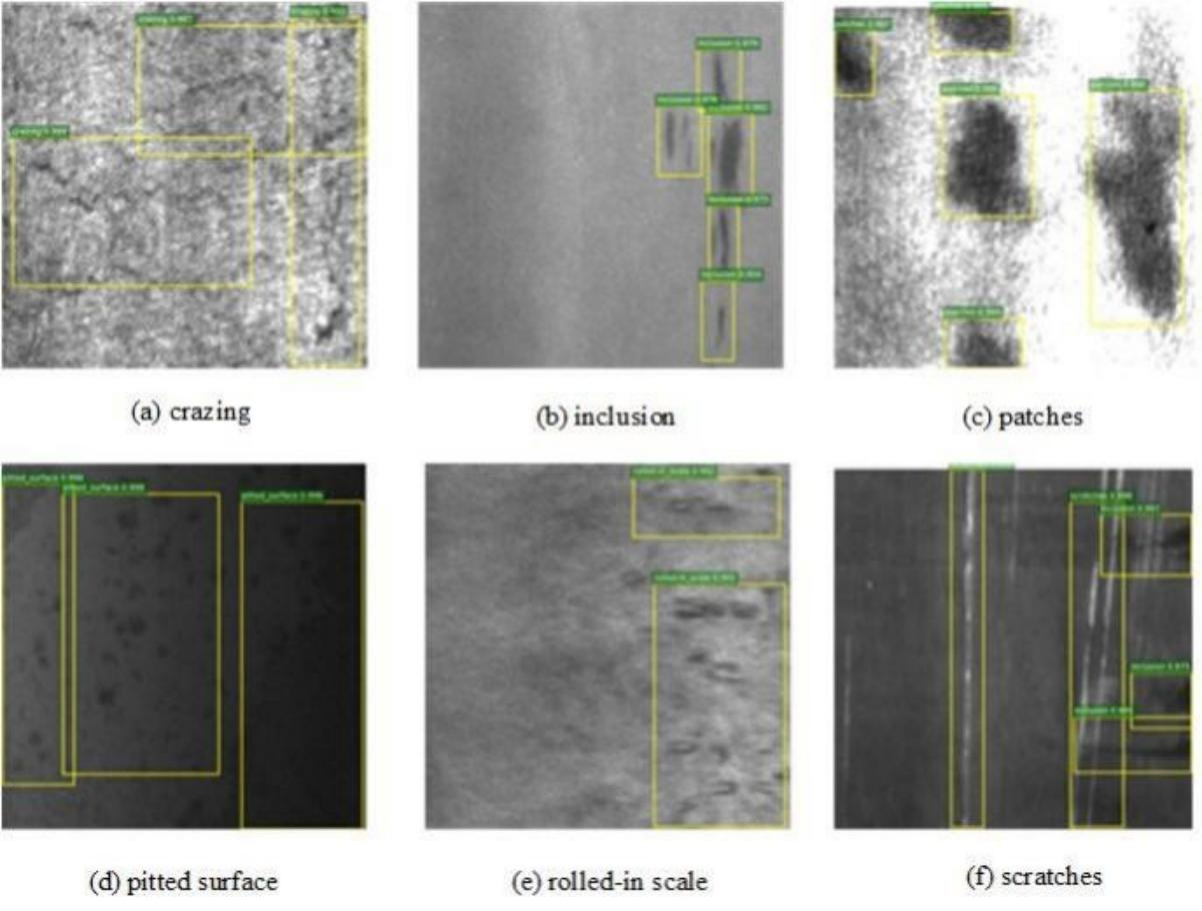
The label of defect category in NEU-DET.

The selected dataset for this paper is NEU-DET, which is a dataset of steel surfaces published by Northeastern University. It consists of six types of defects found on surfaces: Rolled-in Scale, Patches, Crazing, Pitted Surfaces, Inclusions, and Scratches. It includes a total of 1800 grayscale images, with 300 samples for each defect type. The dataset provides annotations that indicate the type and location of each defect. In the annotations, the defect location is marked with a yellow box and the defect category and confidence level are labeled with a green label, as shown in Figure 9.

### 5.2. Image Preprocessing

**Figure 10:**
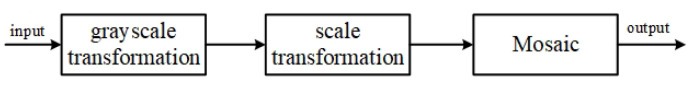
Preprocessing setups of the original dataset.

Before conducting the experiments, we need to perform image preprocessing operations on the original dataset. The specific flow is shown in Figure 10.

As shown in Figure 10, the image preprocessing operation consists of three steps: grayscale transformation, scale transformation, and mosaic. The scale transformation was to change the size of the input image to [224,224], which aimed to reduce the computational complexity of the network, thus shortening the training time of the model. Mosaic is a method of data enhancement, and the main idea is to randomly crop four images and then splice them onto a single image as training data. Its advantages are that it enriches the background of the picture and the four pictures spliced together can increase the batch-size. Furthermore, we also calculated the four pictures when performing batchnormalization. It is not very dependent on its own batch-size, and it can be a good way to save computational resources.

### 5.3. Evaluation Metrics

To assess the effectiveness of the proposed method, AP and mAP, the Average Precision and mean of Average Precision, were used as evaluation metrics for the defect model of steel surfaces. The calculation of AP and mAP is demonstrated in the equations below.

(9) $$P=\frac{TP}{TP+FP}$$

(10) $$R=\frac{TP}{TP+FN}$$

(11) $$AP=\int_{0}^{1}P\left(R\right)dR$$

(12) $$mAP=\frac{1}{N}\sum_{i=1}^{N}AP_{i}$$

The performance of the model can be evaluated using TP (true positive), FP (false positive), and FN (false negative) to represent different types of defects. Precision (*P*) and recall (*R*) are used to measure the accuracy of each type of defect, usually with an IOU of 0.5. *N* represents the total number of defects. The model’s performance is considered better when the values of AP and mAP are closer to 1.

### 5.4. Experimental Environment

The experimental environment of this paper includes Windows 11, NVIDIA GeForce RTX 3060 graphics (Nvidia Corporation, Santa Clara, CA, USA), and a Pytorch 1.12.1 framework. The setting of specific parameters for the training of the model is provided in Table 1.

The settings of hyperparameters in the model during training are shown in Table 2. In the process of model training, “mosaic” and “mixup” were used as data enhancement methods. The probability of this after each step was set to the default value of 0.5. The process of training was divided into a freezing phase and an unfreezing phase. During the freezing phase, the feature extraction network did not change and the system occupied less significant memory to fine-tune the network. “weight-decay” is the coefficient of weight decay, which prevents the model from overfitting.

### 5.5. Ablation Experiment

**Figure 11:**
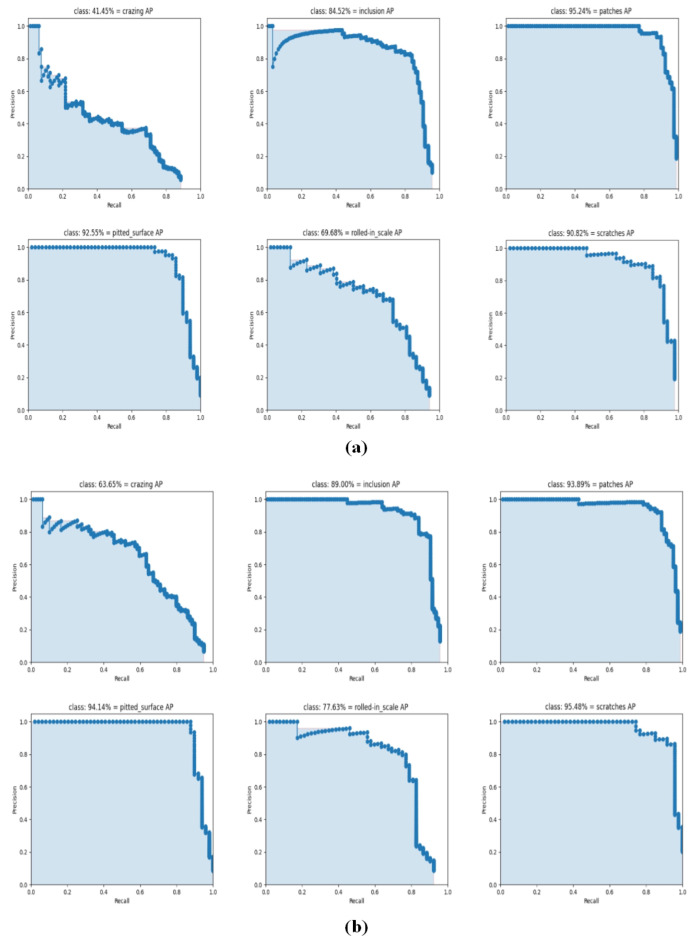
The PR curve of different algorithms: (**a**) the baseline; (**b**) our algorithm.

To assess the effectiveness, the ablation experiments were conducted by combining various improvement schemes with the baseline. Accuracy and PR curves are presented in Table 3 and Figure 11, revealing that both the individual and combined improvement schemes contribute to enhancing the accuracy of defect detection to some degree.

According to Table 3, the In of CBAM in the baseline led to a 2.09 percent improvement in mAP. Similarly, when ASFF was incorporated separately, the mAP improved by 7.06 percent. Furthermore, the addition of CIOU to the baseline resulted in a 6.78 percent increase in accuracy. Obviously, the above results demonstrate that each of three improvements contribute significantly to the defect detection network. Comparative experiments were conducted for all three improvements, and it was observed that the combined approach yielded better results than the individual modules. The final set of experiments combined all three improvements, resulting in a detection accuracy of up to 85.63 percent and an 8.34 percent improvement in mAP compared to the original algorithm.

As depicted in Figure 11, our algorithm demonstrates a significantly superior PR curve compared to the baseline. The AP of our method has also shown improvement to some extent compared to the baseline. Hence, the ablation experiment successfully validates the feasibility and effectiveness of the three improvements proposed in this paper.

### 5.6. Comparative Experiments

**Figure 12:**
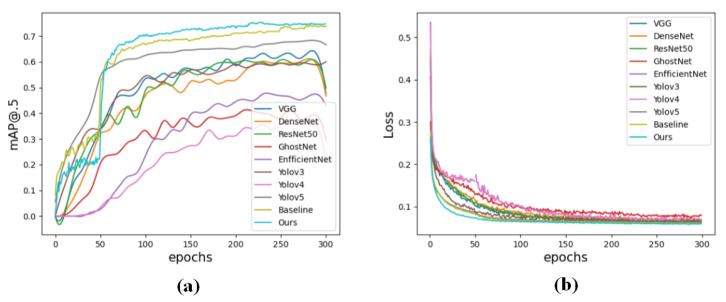
Curves of accuracy and loss as a function of different epochs: (**a**) the curve of mAP; (**b**) the curve of training loss.

In this study, we utilized established deep-learning-based algorithms for object detection to investigate the six mentioned defects. We compared the performance of these different models and present the results of detection and the loss of training components in Table 4 and Figure 12.

According to Table 4, it was observed that several deep learning algorithms (VGG, DenseNet, ResNet50, SSD, Yolov3, and Yolov5) are capable of achieving satisfactory results for defect detection in steel structures. The mAP remains consistently around 70 percent. However, due to the small size of steel surface defects and their corresponding pixels, there are more challenges for these algorithms in accurately recognizing such small-scale defects.

The relationships between the changes in mAP and training loss of different algorithms in the training process are shown in Figure 12. Since the mAP is calculated for the validation set after each epoch of training, it is slightly lower than the final evaluation value. It is easy to notice that our proposed algorithm yields the highest and most stable mAP throughout the training process compared to other major algorithms. Moreover, there are a few algorithms whose mAP decreased near the 300th epoch, which is due to overfitting. Obviously, this indicates that these algorithms are not applicable to this dataset. The loss function converged after 70 epochs of training, while the other main algorithms require multiple iterations to achieve convergence. The results indicate that the improved algorithm converges faster and is more effective in optimizing the model parameters.

The results presented in Table 4 demonstrate that our method achieves precise identification of small-scale defects with a high mAP of 85.63 percent. It indicates an improvement of 8.34 percent in accuracy compared to the baseline. Furthermore, compared to other major algorithms, it performs exceptionally well on the NEU-DET dataset. Additionally, the baseline outperforms other networks with a significantly higher AP of 95.24 percent on the Pa defect.

The experiments demonstrate that our method for defect detection in steel structures significantly improves the accuracy compared to the baseline and other networks. This paper provides evidence of the superiority of our algorithm.

## 6. Conclusions

In this study, we investigated the defect detection of steel structures and utilized an open-source dataset to conduct experiments. Firstly, to address the issue of small-scale defect images, CBAM was proposed as an enhancement to the baseline model. CBAM improved the extraction of surface information of deep features and enhanced the model’s ability to learn sample defects. Secondly, ASFF was introduced to make the model adaptively learn weight information from different scale feature maps, thereby increasing the recognition rate of positive example samples. Finally, the loss of CIOU was used to address the problem of the scale consistency of the bounding box and expedite the convergence time of the whole model. The experiments demonstrate that our method exhibits a notable improvement in the accuracy of defect detection, highlighting its superiority when compared to the baseline.

The methods in this work have improved the mAP to some extent, but the study did not consider the computational efficiency of the model. We found that the training time of the algorithm proposed in this work was almost the same as that of the baseline during our comparison experiments. Furthermore, the specific hardware environment is shown in Table 1. Methods that achieve a trade-off between the accuracy and computational efficiency of defect detection are also a future research interest. After that, we will consider using a lightweight feature extraction network instead of a backbone network to reduce the training time of the model.

The selected steel structure samples for this experiment are too homogeneous, which inevitably leads to the problem of poor adaptation of the model. Therefore, future research will include other types of metal defects. Considering the time consumed by the current algorithm, future research will focus on designing a lightweight neural network that balances the accuracy and time of detection.

## Figures and Tables

**Table 1:** Configuration of experimental parameters.

Setting	Parameters
CPU	Intel Core i7-12700H (Intel Corporation, Santa Clara, CA, USA)
GPU	NVIDIA RTX 3060 Laptop
System	Windows 11
Pytorch	Torch 1.12
IOU	0.5
Epoch	300
Batch size	8
Optimizer	SGD

**Table 2:** Hyperparameter settings for training the model.

Parameter	Value
num-classes	6
input-shape	[224,224]
mosaic-prob	0.5
mixup-prob	0.5
special-avg-ratio	0.7
max-epoch	300
freeze-batch-size	16
unfreeze-batch-size	8
learning-rate	0.01
momentum	0.937
weight-decay	0.0005
num-workers	2

**Table 3:** Results of ablation experiment.

Methods			AP (%)						mAP
CBAM	ASFF	CIOU	Cr	In	Pa	Ps	Rs	Sc	(%)
			41.45	84.52	**95.24**	92.55	69.68	90.82	79.04
*√*			42.03	82.90	93.35	**95.54**	72.15	**98.15**	80.69
	*√*		58.59	87.39	93.14	93.44	77.89	97.30	84.62
		*√*	58.89	88.78	94.80	94.86	72.09	96.99	84.40
*√*	*√*		61.12	87.09	93.68	94.13	**79.61**	97.05	85.45
*√*		*√*	63.53	**89.91**	93.66	92.40	74.96	95.71	85.03
	*√*	*√*	**67.08**	86.43	93.94	92.95	74.91	95.59	85.08
*√*	*√*	*√*	63.65	89.00	93.89	94.14	77.63	95.48	**85.63**

**Table 4:** Comparative experiments of different algorithms.

Algorithm	AP (%)						mAP (%)
	Cr	In	Pa	Ps	Rs	Sc	
**VGG**	30.73	79.52	93.15	86.98	61.01	89.06	73.41
**DenseNet**	31.18	73.96	73.71	82.27	49.90	85.81	69.47
**GhostNet**	12.98	66.29	76.02	78.76	31.21	59.73	54.16
**ResNet50**	29.68	78.92	91.36	84.42	53.95	85.56	70.65
**SSD**	33.30	80.18	94.74	88.43	64.68	80.22	73.59
**EnfficientNet**	21.30	70.04	83.51	77.46	44.71	65.61	60.44
**Yolov3**	25.98	75.48	89.94	85.43	60.39	88.12	70.89
**Yolov5**	34.75	81.81	90.92	86.98	66.09	90.50	75.17
**Baseline**	41.45	84.52	**95.24**	92.55	69.68	90.82	79.04
**Ours**	**63.65**	**89.00**	93.89	**94.14**	**77.63**	**95.48**	**85.63**
